# Improvement of oxidative stability and textural properties of fermented sausage via addition of pistachio hull extract

**DOI:** 10.1002/fsn3.1594

**Published:** 2020-05-16

**Authors:** Seyede Saba Lashgari, Zohre Noorolahi, Mohamad Ali Sahari, Hassan Ahmadi Gavlighi

**Affiliations:** ^1^ Department of Food Science and Technology Faculty of Agriculture Tarbiat Modares University Tehran Iran

**Keywords:** antioxidant and antimicrobial activities, fermented sausage, lipid oxidation, pistachio hull extract

## Abstract

The purpose of this study was to evaluate the effectiveness of pistachio hull extract (PHE) as an antioxidant and antimicrobial agent for preservation of dry fermented sausages during fermentation and storage period. Sausages were prepared using starter culture (Biobak K) and treated with three levels of PHE (500, 750 and 1,000 ppm). The results showed that PHE at concentrations of 500 ppm and 750 ppm decreased significantly (*p* < .05) the TBARS content of the sausage samples compared to control (without PHE). Moreover, PHE increased L^*^ and a^*^ value of samples during fermentation period but did not affect the color of samples during storage period. The PHE was also able to improve the chewiness and gumminess of the fermented sausage. Evaluation of microbial properties (total viable count, yeast and molds, lactic acid bacteria, staphylococci and *Enterobacteriaceae*) also showed that antimicrobial activity of PHE in fermented sausage.

## Graphical abstract

The purpose of this study was to evaluate the effectiveness of pistachio hull extract (PHE) as an antioxidant and antimicrobial agent for preservation of dry fermented sausages during fermentation and storage period. Evaluation of microbial properties (total viable count, yeast and molds, lactic acid bacteria, staphylococci and Enterobacteriacea) also showed that antimicrobial activity of PHE in fermented sausage.

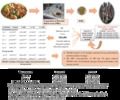

## 1 INTRODUCTION

Fermented sausages are produced as a result of biochemical, microbiological, physical, and organoleptic changes on meat during the ripening stage under defined temperature and humidity conditions (Essid & Hassouna, 2013). In fact, remarkable changes in the odor, taste, organoleptic characteristics, and shelf‐life of raw meat, which are mainly caused by lactic acid bacteria, lead to production of fermented sausages (Dertli et al., 2016).

Fermented sausages are uncooked products, so they are exposed to pathogenic and spoilage bacteria and lipid oxidation, resulting in reduced their shelf‐life (Van Ba et al., 2016). The addition of antioxidants has become popular as a means of increasing the shelf‐life and reducing the nutrient losses of meat products by inhibiting or delaying oxidation (Rajaei, Barzegar, & Sahari, 2010). The use of synthetic antioxidants (e.g., BHA, BHT, and PG) is suspected to cause toxicity problems that negatively affect the consumers’ health. Therefore, a new trend to substitute natural sources of antioxidants (e.g., plant and herb extracts) instead of synthetic ones has received the most attention by consumers and meat processors (Van Ba et al., 2016). Previous studies have shown the antioxidant activities of grape seed and chestnut extracts (Lorenzo, Gonzalez‐Rodriguez, & Amado, 2013), edible mushroom extract (Van Ba et al., 2016), oregano and thyme essential oils (Adab & Hassouna, 2016), and rose polyphenols (Zhang et al., 2017) in fermented sausage.

The waste material produced by the agricultural industry causes environmental problems. Since these materials often contain many polyphenolic compounds, the possibility of using them as antioxidant compounds in the food industry can be an important step in maintaining environmental balance (Lorenzo, Sineiro, Amado, & Franco, 2014). Endocarp (hull), branch, leaf, and bark are considered as by‐products of fresh pistachio industry so that the endocarp is the most abundant of them (60%). Previous studies have shown the high antioxidant activity of pistachio hull (Barreca et al., 2016; Bellocco et al., 2016; Goli, Barzegar, & Sahari, 2005; Martorana et al., 2013; Rajaei et al., 2010). Gallic acid, catechin, cyanidin‐3–0‐galactoside, oridicctol‐7–0‐glycoside, and epicatechin appear to be responsible for the antioxidant properties of pistachio hull (Tomaino et al., 2010). In addition, the endocarp extract has antimicrobial activity against pathogenic Gram‐positive bacteria (Rajaei et al., 2010).

The purpose of this study was to use the PHE as a low‐cost bioactive source in fermented sausage processing and identifying some characteristics of this product. Furthermore, the ability of PHE to maintain chemical and microbial quality of fermented sausage during the fermentation and storage period has been evaluated.

## 2 MATERIALS AND METHODS

### 2.1 Plant material and chemicals

Ahmad Aghaei pistachio endocarp was obtained from Kerman Agricultural Research Center, Kerman Province, Iran. Starter culture (Biobak K) was purchased from (Wiberg‐Salzburg, Austria), containing *Lactobacillus sake*, *Staphylococcus xylosus*, *Staphylococcus carnosus,* and *Pediococcus pentosaceus*. Butylated hydroxytoluene (BHA), perchloric acid 70%–72%, ethanol, sodium chloride, n‐hexane, copper sulfate, potassium sulfate, selenium, chloride acid, Plat Count Agar, MRS agar, Mannitol Salt Agar, Sabouraud Dextrose Agar, and Violet Red Bile Glucose Agar were purchased from the Merck Company of Germany.

### 2.2 Preparation of pistachio hull extract (PHE)

Pistachio endocarp powder was mixed with distilled water with a ratio of 1:15 and stirred for 8 hr at 25 ºC. Subsequently, it was centrifuged for 10 min at 3000*g*, and then, the supernatant was passed through Whatman No. 42. The extract was placed in vacuum at 40°C for 8 to 12 hr for concentration and then transferred to the freeze dryer. After drying, the extracted powder was stored in an airtight container at −20°C until the test was carried out.

### 2.3 Formulation and preparation of fermented sausage

Sausage dough was prepared by mixing 75% beef (fat‐less and without antibiotics) and 25% beef fat. After adding salt (14 g/kg), sodium nitrite (0.1 g/kg), paprika spices (36 g/kg), glucose (15 g/kg), starter culture (Biobak K), and PHE (500, 750, and 1,000 ppm), the compounds were completely mixed and stuffed into the natural casting. The fermentation step was conducted at 23°C and the relative humidity of 85 to 95% for 5 days followed by drying stage at 14°C and the relative humidity of 75% to 80% for 23 (Essid & Hassouna, 2013). After ripening, the produced samples (five samples from each treatment) were packed in plastic bag, sealed and stored at 4°C for 60 days. Chemical tests had three replicates on 0, 7, 14, 21, 28, 60, and 90 days, and microbial tests were performed in three replications on 0, 28, 60, and 90 days.

### 2.4 Analytical methods

#### 2.4.1 pH, moisture, water activity, TBARS index, and color evaluation

The pH of sausages was measured using a digital pH meter (Metrohm model, pH Lab 827). For this purpose, 1 g of each sample was weighed and homogenized for 10 min after adding 10 ml of distilled water.

Moisture percentage was determined by oven drying (Memmert ULM500, Germany) and using the method of AOAC (1997). Water activity of samples was evaluated using the AW SPRINT TH‐500 device (Pfäffikon, Switzerland) at 25°C.

Measuring the TBARS index was performed using Salih, Smith, Price, and Dawson (1987). TBARS values were calculated against a standard curve (prepared by 1,1,3,3 tetraethoxypropane) and expressed as mg malonaldehyde/kg.

Color was determined at the cut surface of each sample using a Hunter laboratory colorimeter (Color Flex, Virginia, USA) by reading the L^*^ (brightness), a^*^ (redness), and b^*^ (yellowness) factors after calibration of the device with black and white plates (L^*^ = 92.23, a^*^ = −1.29, and b^*^ = 1.19).

#### 2.4.2 Microbial analysis

For microbiological analysis, 10 g of each sample was aseptically weighted in a sterile plastic bag (after removing the casing). Subsequently, the samples were homogenized with 90 ml sterile solution of 0.1% (w/v) peptone water. Total viable counts (TVC) were enumerated by Plate Count Agar (Merck) after incubating at 30°C for 48 hr, lactic acid bacteria by Man Rogosa Sharpe agar (Merck) after incubating at 30°C for 48 hr days, staphylococci by Mannitol Salt Agar (Merck) after incubating at 37°C for 37 hr yeasts, and molds by Sabouraud Dextrose Agar (Merck) after incubating at 25°C for 5 days and *Enterobacteriacea* by Violet Red Bile Glucose Agar (Merck) after incubating at 37°C for 24 hr. Plates with 30–300 colonies were counted. The microbiological data were transformed into logarithms of the number of colony‐forming units (CFU/g).

#### 2.4.3 Texture profile evaluation

The texture of sausage samples was evaluated by Brookfield Texture Analyzer equipped with a TA25/ 1,000 cylindrical probe with 50 mm diameter, and Load Cell 100 N, according to the method of Dertli et al. (2016). Sausage pieces of 1 × 1 × 2.5 cm (height × width × length) were compressed at a cross‐head speed of 2 mm/s. The capacity of load cell was 5 kg, and its time interval was set at 30 s between the two compression cycles. Hardness (kg), cohesiveness, springiness (mm), gumminess (kg), and chewiness were obtained. The final results are the average of at least three reproducible runs for each treatment.

#### 2.4.4 Sensorial evaluation

At the end of storage time, the samples were evaluated by 30 semi‐trained evaluators (consisting of 12 men and 18 women) from the Department of Food Science and Technology of Tarbiat Modares University. The samples were cut in slices about 5 mm thickness after removing their casing and then served at room temperature on white plastic dishes. A continue scale between among 1 and 5 was used for evaluation odor (1 = extreme off‐odor, 2 = moderate off‐odor, 3 = small off‐odor, 4 = slight off‐odor, and 5 = no off‐odor); flavor (1 = extreme sour flavor, 2 = moderate sour flavor, 3 = small sour flavor, 4 = slight sour flavor, and 5 = no sour flavor); overall acceptability (1 = extremely undesirable, 3 = moderately undesirable, 3 = undesirable, 4 = moderately desirable, and 5 = extremely desirable). Water was used to clean the palates and remove residual flavors, at the beginning of the session and in between samples (Ciriano et al., 2009).

### 2.5 Statistical analysis

The number of examined samples was 140 (including 35 control samples and 105 samples with extracts at three levels), and sampling was done in 0, 7, 14, 21, 28, 60, and 90 days. Data analysis was performed using one‐way ANOVA with keeping each factor constant, by the SPSS software (ver. 21) in a completely randomized design. For sensory evaluation data, all the panelists were included in the sensory evaluation portion of analysis. To investigate significant differences between the means (*p* < .05) as a result of factors, Duncan test was used. All the results are shown in terms of mean of three replicates ± standard deviation.

## 3 RESULTS AND DISCUSSION

### 3.1 pH evaluation of sausage samples

Variations of pH in fermented sausages during fermentation and storage are showed in Table 1. The pH of all samples decreased sharply from day 1 to 7. The decrease of pH during the first week of fermentation is due to the decomposition of carbohydrates and accumulation of organic acids (such as lactic acid) by the LABs present in the starter culture (Van Ba et al., 2016). The pH value of all samples did not significantly (*p* < .05) changed from day 7 until the end of fermentation period but it was increased in control and 500‐E samples during the first month of storage. Moreover, the pH value of 700‐E and 1000‐E samples increased after two month of storage. The pH increase during storage is attributed to production of amines, peptides, and amino acids from proteolysis reactions and reduction of lactic acid production compared to the formation of low molecular weight nitrogen compounds (Kurćubić et al., 2014). In day 28 (end of ripening) and 60 (first month of storage), control sample had the lowest of pH values and there was no significant (*p* < .05) difference between the pH values of 500‐E, 750‐E, and 1000‐E samples.

**Table 1:** pH variations of sausage samples during fermentation and storage period

Treatment	Control	E‐500	E‐750	E‐1000
Fermentation period				
day 1	6.13 ± 0.02^aA^	6.02 ± 0.03^bA^	6.13 ± 0.01^aA^	6.02 ± 0.01^bA^
day 7	4.74 ± 0.06^bC^	4.9 ± 0.1^abC^	4.94 ± 0.05^aC^	4.99 ± 0.07^aC^
day 14	4.77 ± 0.02^aC^	5.24 ± 0.09^bB^	4.84 ± 0.2^bC^	5.2 ± 0.09^aB^
day 21	4.75 ± 0.03^bC^	4.97 ± 0.05^aC^	5.06 ± 0.16^aC^	5.04 ± 0.04^aBC^
day 28	4.74 ± 0 .07^bC^	5.04 ± 0.02^aC^	4.99 ± 0 .08^aC^	5.09 ± 0.12^aBC^
Storage period				
day 60	4.85 ± 0.04^bB^	5.28 ± 0.04^aB^	4.96 ± 0.12^bC^	5.00 ± 0.14b^C^
day 90	4.73 ± 0.06^cC^	4.98 ± 0.04^bC^	5.72 ± 0.2^aB^	5.11 ± 0.12b^BC^

Note

Results are expressed as means ± *SD*; different lowercase letters represent a significant difference in each row, and capital letters indicate a significant difference in each column (*p* < .05).

Control: sample without any extract, E‐500: sample with 500 ppm of extract, E‐750: sample with 750 ppm of extract, and E‐1000: sample with 1,000 ppm of extract.

### 3.2 Moisture evaluation of sausage samples

Moisture content of fermented sausage samples during fermentation and storage period (90 days) are shown in Table 2. Moisture content of all samples decreased significantly (*p* < .05) during fermentation period. This decrease was due to the loss of moisture at high temperature and low relative humidity percentage of this period (Pateiro, Bermúdez, Lorenzo, & Franco, 2015). During storage period, moisture content did not change significantly in all sausages (except E‐750), probably due to the impact of the packaging on preventing of moisture loss (Kurćubić et al., 2014). According to Table 2, PHE increased the moisture content of sausages during fermentation and storage periods so that E‐1000 had the highest amount of moisture and was not significantly different from the E‐750. Some previous studies also showed that the use of natural extracts increased the moisture content of fermented sausages (Lorenzo et al., 2013; Pateiro et al., 2015).

**Table 2:** Moisture and water activity variations of sausage samples during fermentation and storage period

Treatment	Control	E‐500	E‐750	E‐1000
Fermentation period				
Day 1				
Moisture	58.31 ± 0.62^aA^	60.38 ± 0.93^aA^	59.59 ± 0.20^aA^	66.45 ± 0.23^aA^
a_w_	0.899 ± 0.003^bA^	0.905 ± 0.015^abA^	0.925 ± 0.004^aA^	0.908 ± 0.016^abA^
Day 7				
Moisture	43.63 ± 0.80^abB^	37.66 ± 0.86^bB^	45.32 ± 0.49^aB^	46.51 ± 0.99^aB^
a_w_	0.851 ± 0.009^bB^	0.884 ± 0.021^abA^	0.864 ± 0.014^aB^	0.877 ± 0.013^cB^
Day 14				
Moisture	35.08 ± 0.40^bC^	32.08 ± 39^bC^	41.42 ± 0.50^aB^	43.33 ± 30^aB^
a_w_	0.833 ± 0.010^aB^	0.836 ± 0.017^aB^	0.847 ± 0.010^aB^	0.843 ± 0.006^aC^
Day 21				
Moisture	30.3 ± 86^bC^	21.97 ± 58^cD^	32.97 ± 59^abC^	34.93 ± 81^aC^
a_w_	0.738 ± 0.008^bC^	0.782 ± 0.011^abC^	0.806 ± 0.012^aC^	0.775 ± 0.012^bD^
Day 28				
Moisture	20.57 ± 46^bD^	18.70 ± 0.10^bDE^	29.12 ± 92^aC^	29.16 ± 0.55^aCD^
a_w_	0.718 ± 0.010^bC^	0.736 ± 0.015^bD^	0.785 ± 0.006^aCD^	0.746 ± 0.023^bDE^
Storage period				
Day 60				
Moisture	21.14 ± 0.30^abD^	16.93 ± 0.93^bE^	22.08 ± 0.48^abD^	26.20 ± 80^aD^
a_w_	0.673 ± 0.003^bD^	0.681 ± 0.012^bE^	0.764 ± 0.020^aD^	0.733 ± 0.025^aEF^
Day 90				
Moisture	20.38 ± 0.41^bD^	16.51 ± 0.74^cE^	28.55 ± 0.56^aCD^	28.96 ± 0.64^aCD^
a_w_	0.679 ± 0.023^bcD^	0.658 ± 0.029^cE^	0.803 ± 0.021^aC^	0.711 ± 0.014^bF^

Note

Results are expressed as means ± standard deviation; different lowercase letters represent a significant difference in each row, and different capital letters indicate a significant difference in each column for each parameter (*p* < .05).

Control: sample without any extract, E‐500: sample with 500 ppm of extract, E‐750: sample with 750 ppm of extract, and E‐1000: sample with 1,000 ppm of extract.

Water activity (a_w_) variations of samples during fermentation and storage period were similar to moisture content variations of them (Table 2). Application of PHE in fermented sausage increased the a_w_ amount compared to control sample, but the concentration of PHE had no significant effect on this parameter. According to this result, Aquilani et al. (2018) also showed that antioxidants from grape seed and chestnut had no effect on a_w_ amounts of fermented sausages.

### 3.3 Color evaluation of sausage samples

Color is one of the most important parameters of sausage that attracts consumers. The color characteristics (L^*^, a^*^, and b^*^) of fermented sausages during fermentation and storage period are presented in Table 3. The L* value of all samples decreased sharply during the first week of fermentation. This decrease could be attributed to the high moisture losses of samples (Bozkurt & Bayram, 2006; Lorenzo et al., 2013; Lorenzo, Temperán, Bermúdez, Cobas, & Purriños, 2012). No significant changes were observed in L* of all samples during storage. At the end of fermentation period (28th day), the highest L* value was observed in E‐500 sample which had no significant difference with E‐750 sample. Moreover, there were no significant differences between the L* values of all samples at the end of storage period.

**Table 3:** Color characteristics of sausage samples during fermentation and storage period

Treatment	Control				E−500				E−750				E−1000		
	L^*^	a^*^	b^*^	L^*^		a^*^	b^*^	L^*^		a^*^	b^*^	L^*^		a^*^	b^*^
Fermentation period															
Day 1	41.1 ± 3.2^aA^	17.8 ± 0.7^bA^	13 ± 1.5^bA^	40.8 ± 2.1^aA^		21.3 ± 2.6^abA^	17.3 ± 2.5^aA^	37 ± 3.8^aA^		24 ± 0.1^aA^	20.4 ± 0.3^aA^	34.2 ± 2.3^aA^		20.7 ± 2.8^abA^	17.6 ± 2.0^aA^
Day 7	34.5 ± 2.1^aBC^	17.8 ± 0.7^aA^	12.4 ± 1.9^aA^	33.1 ± 1.1^abCD^		18.1 ± 0.9^aB^	13.8 ± 1.6^aB^	32.6 ± 0.5^abBC^		16.8 ± 1.7^aB^	12.7 ± 1.4^aB^	30.8 ± 1.3^bD^		18.5 ± 0.9^aAB^	13.6 ± 1.5^aB^
Day 14	35.8 ± 0.5^aB^	17.8 ± 0.2^aA^	12.5 ± 0.8^aA^	37.7 ± 1.2^aB^		17.5 ± 0.3^bBC^	12.5 ± 0.1^aBC^	37.5 ± 0.1^aA^		17.0 ± 0.1^bB^	12.1 ± 0.2^aBC^	35.8 ± 2.6^aB^		16.4 ± 0.8^aBC^	11.5 ± 0.8^aBC^
Day 21	33.4 ± 1.9^aBC^	15.5 ± 1.0^aB^	9.6 ± 1.7^aB^	34.8 ± 0.6^aC^		14.9 ± 0.9^aD^	10.5 ± 0.7^aD^	35.5 ± 3.7^aAB^		15.8 ± 0.3^aB^	12.0 ± 0.9^aC^	33.4 ± 1.7^aC^		16.0 ± 2.2^aBC^	10.7 ± 2.3^aCD^
Day 28	32.4 ± 0.1^bBC^	14.8 ± 0.0^bB^	10.0 ± 0.1^bB^	35.8 ± 0.9^aBC^		15.6 ± 0.4^aCD^	12.3 ± 0.8^aBC^	35.2 ± 0.6^aAB^		15.6 ± 0.2^aB^	11.0 ± 0.4^abC^	32.4 ± 0.7^bCD^		14.7 ± 0.6^bC^	10.3 ± 1.1^bCD^
Storage period															
Day 60	30.8 ± 0.1^aC^	11.1 ± 1.5^aC^	8.1 ± 1.3^aB^	28.4 ± 2.0^aE^		10.7 ± 1.1^aE^	8.9 ± 0.6^aD^	31.2 ± 2.1^aC^		11.5 ± 1.0^aC^	8.8 ± 1.2^aD^	28.0 ± 4.1^aE^		10.3 ± 1.4^aD^	8.0 ± 1.5^aD^
Day 90	30.6 ± 0.4^abC^	11.5 ± 1.1^aC^	8.1 ± 0.7^bC^	30.1 ± 1.6^abDE^		11.1 ± 1.4^aE^	9.9 ± 0.6^aD^	32.2 ± 1.2^aBC^		11.9 ± 0.9^aC^	8.3 ± 0.6^bD^	27.6 ± 3.1^bE^		11.6 ± 1.5^aD^	8.4 ± 0.6^bD^

Note

Results are expressed as means ± standard deviation; different lowercase letters represent a significant difference in each row, and capital letters indicate a significant difference in each column (*p* < .05).

Control: sample without any extract, E‐500: sample with 500 ppm of extract, E‐750: sample with 750 ppm of extract, and E‐1000: sample with 1,000 ppm of extract.

The amount of a* value of all samples decreased significantly during fermentation period and first month of storage and then remained constant at the second month of storage (Table 3). Reduced a* value can be attributed to partial or total nitrosomyoglobin denaturation caused by production of lactic acid (Lorenzo et al., 2013). At the end of ripening period, the highest amount of a^*^ value was observed in E‐500 and E‐750 samples so that a^*^ values of these samples were higher than control sample. This suggested the protective effect of PGE on nitrosomyoglobin oxidation (Fernandes, Trindade, Lorenzo, Munekata, & de Melo, 2016). Previous studies have also shown the effect of natural extracts on improving a^*^ value of fermented sausages (Lorenzo et al., 2013; Pateiro et al., 2015).

The amount of b^*^ value in control, E‐500, E‐750, and E‐1000 samples was significantly (*p* < .05) decreased during the fermentation and storage period (Table 3) that indicated color of all sausages turned to blue rather than yellow (Karabacak & Bozkurt, 2008). Reduction of b^*^ value can be attributed to the production of brown melanoidins due to the browning reactions (Bozkurt, 2006). The results showed that PHE had no significant effect on b^*^ value of fermented sausages that was accordance with Karabacak and Bozkurt (2008) and Bozkurt (2006).

### 3.4 Texture evaluation of sausage samples

Textural properties of fermented sausage samples (hardness, cohesion, chewing ability, elasticity, and gummy state) in the 28th day of fermentation are given in Table 4. E‐500 sample had no significant (*p* > .05) difference with control sample in all textural parameters but the chewiness of E‐750 sample was lower than control sample. The high chewiness value of control sample indicated that this sample was tougher than E‐750 sample. Similar to this result, Lorenzo et al. (2013) also showed that the use of natural extract reduced the amount of chewiness. According to Table 4, hardness of E‐1000 sample was higher than control sample, whereas previous studies showed that the use of natural extracts reduced hardness of fermented sausages (Lorenzo et al., 2013; Pateiro et al., 2015). This result was probably due to the lower moisture content and a_w_ of control sample compared to E‐1000 sample.

**Table 4:** Textural characteristics of sausage samples in the 28th day of fermentation

Treatment	Control	E‐500	E‐750	E‐1000
Hardness (kg)	16.42 ± 3.9^a^	19.33 ± 9.0^ab^	13.53 ± 4.2^a^	30.1 ± 4.3^b^
Cohesiveness	0.40 ± 0.1^a^	0.34 ± 0.25^a^	0.53 ± 0.02^a^	0.49 ± 0.01^a^
Springiness (mm)	2.90 ± 0.28^a^	2.93 ± 0.53^a^	3.22 ± 0.22^a^	2.96 ± 0.17^a^
Gumminess (kg)	10.12 ± 4.1^ab^	8.34 ± 3.1^ab^	6.21 ± 2.3^b^	14.75 ± 2.18^a^
Chewiness	31.73 ± 8.2^b^	25.23 ± 6.8^bc^	18.98 ± 2.37^c^	43.4 ± 3.9^a^

Note

Results are expressed as means ± *SD*; different lowercase letters represent a significant difference in each row (*p* < .05).

Control: sample without any extract, E‐500: sample with 500 ppm of extract, E‐750: sample with 750 ppm of extract, and E‐1000: sample with 1,000 ppm of extract.

### 3.5 TBARS evaluation of sausage samples

TBARS variations of the sausage samples during fermentation and storage period are presented in Table 5. As shown, TBARS amount of all samples increased significantly (*p* < .05) during fermentation and first month of storage. The increase of TBARS value in meat products is due to lipid oxidation and dehydration of samples (Fan, Yi, Zhang, & Diao, 2015; Pelser, Linssen, Legger, & Houben, 2007; Zanardi, Ghidini, Battaglia, & Chizzolini, 2004).TBARS amount of E‐500 sample increased continuously from day 60 to day 90, whereas this value decreased in control sample probably due to reaction of malondialdehyde with proteins and sugars (Ansorena & Astiasarán, 2004). The PHE (concentrations of 500 and 750 ppm) reduced TBARS value of fermented sausages during fermentation and storage periods (Table 5). This result indicated that the PHE was able to inhibit oxidation of fermented sausages due to high antioxidant activity. According to this result, Zhang et al. (2017) also showed that the use of rose polyphenols reduced TBARS value of fermented sausages. The addition of grape seed and chestnut to fermented sausages also decreased the oxidation of samples (Lorenzo et al., 2013).

**Table 5:** TBARS variations of sausage samples during fermentation and storage period (mg malonaldehyde/kg)

Treatment	Control	E‐500	E‐750	E‐1000
Fermentation period				
Day 1	1.21 ± 0.02^aE^	1.18 ± 0.08^aD^	1.13 ± 0.06^aC^	1.21 ± 0.09^aC^
Day 7	1.43 ± 0.2^aDE^	1.02 ± 0.02^bcD^	0.84 ± 0.16^cD^	1.28 ± 0.17^abC^
Day 14	1.52 ± 0.09^aCDE^	1.22 ± 0.07^bCD^	1.17 ± 0.04^bBC^	1.42 ± 0.05^aC^
Day 21	1.70 ± 0.19^aCD^	1.47 ± 0.12^abC^	1.42 ± 0.05^bBC^	1.41 ± 0.03^bC^
Day 28	1.94 ± 0.18^aC^	1.46 ± 0.17^bC^	1.44 ± 0.06^bB^	1.83 ± 0.18^aBC^
Storage period				
Day 60	3.96 ± 0.51^aA^	2.86 ± 0.38^bB^	2.87 ± 0.11^bA^	2.88 ± 0.27^bA^
Day 90	3.14 ± 0.17^abB^	3.40 ± 0.19^aA^	2.93 ± 0.21^abA^	2.68 ± 0.33^bA^

Note

Results are expressed as means ± standard deviation; different lowercase letters represent a significant difference in each row, and capital letters indicate a significant difference in each column (*p* < .05).

Control: sample without any extract, E‐500: sample with 500 ppm of extract, E‐750: sample with 750 ppm of extract, and E‐1000: sample with 1,000 ppm of extract.

### 3.6 Microbial evaluation of sausage samples

Microbial count of sausage samples during fermentation and storage period is presented in Table 6. It is worth noting that *Enterobacteriaceae* was not detectible in all samples during fermentation and storage periods, so these results were not reported.

**Table 6:** Microbial analysis of sausage samples during fermentation and storage period

Treatment	Total microorganisms				Lactic acid bacteria				Yeast and molds				Staphylococci			
	Control	E‐500	E‐750	E‐1000	Control	E‐500	E‐750	E‐1000	Control	E‐500	E‐750	E‐1000	Control	E‐500	E‐750	E‐1000
Fermentation period																
Day 1	4.9 ± 0.1^aC^	5.3 ± 0.5^aA^	5.4 ± 0.4^aB^	5.4 ± 0.4^aB^	4.9 ± 0.0^aC^	5.3 ± 0.4^aB^	5.2 ± 0.1^aB^	5.2 ± 0.1^aB^	4.9 ± 0.1^bC^	5.2 ± 0.4^abA^	5.5 ± 0.2^aB^	5.3 ± 0.1^abB^	4.5 ± 0.1^aA^	4.4 ± 0.3^aB^	4.4 ± 0.2^aB^	4.4 ± 0.3^aB^
Day 14	5.3 ± 0.1^abB^	5.4 ± 0.2^abA^	5.7 ± 0.4^aB^	4.9 ± 0.4^bB^	5.2 ± 0.1^aBC^	5.7 ± 0.2^aAB^	5.5 ± 0.5^aB^	5.4 ± 0.4^aB^	5.3 ± 0.1^aB^	5.3 ± 0.2^aA^	5.7 ± 0.4^aB^	5.2 ± 0.4^aB^	4.0 ± 0.1^bAB^	4.2 ± 0.4^abBC^	4.7 ± 0.1^aAB^	4.4 ± 0.3^abB^
Day 28	6.0 ± 0.2^bA^	5.6 ± 0.1^cA^	6.5 ± 0.1^aA^	4.9 ± 0.2^dB^	5.8 ± 0.5^bAB^	5.8 ± 0.1^bA^	6.4 ± 0.1^aA^	5.3 ± 0.1^cB^	6.1 ± 0.2^bA^	5.6 ± 0.1^cA^	6.5 ± 0.1^aA^	5.1 ± 0.3^dB^	3.5 ± 0.2^cB^	3.8 ± 0.6^cC^	5.0 ± 0.0^aA^	4.3 ± 0.5^bB^
Storage period																
Day 60	^Bb^1.0 ± 3.5	5. 8 ± 0.7^bA^	5.9 ± 0.3^abB^	6.6 ± 0.1^aA^	6.0 ± 0.2^bA^	5.7 ± 0.2^bcA^	6.4 ± 0.1^aA^	6.3 ± 0.0^aA^	4.5 ± 0.3^cD^	5.3 ± 0.8^bcA^	5.6 ± 0.5^abB^	6.5 ± 0.1^aA^	4.3 ± 0.7^bA^	4.6 ± 0.2^bB^	3.8 ± 0.3^cC^	5.2 ± 0.1^aA^
Day 90	6.1 ± 0.1^bcA^	5.9 ± 0.2^cA^	6.5 ± 0.1^aA^	6.3 ± 0.1^abA^	5.3 ± 0.7^bABC^	5.9 ± 0.2^abA^	5.4 ± 0.1^bB^	6.5 ± 0.1^aA^	5.4 ± 0.1^cB^	5.7 ± 0.5^bcA^	6.4 ± 0.1^aA^	6.2 ± 0.1^abA^	4.0 ± 0.5^cAB^	5.6 ± 0.6^aA^	4.8 ± 0.1^bA^	5.4 ± 0.1^abA^

Note

Results are expressed as means ± standard deviation; different lowercase letters represent a significant difference in each row, and capital letters indicate a significant difference in each column (*p* < .05).

Control: sample without any extract, E‐500: sample with 500 ppm of extract, E‐750: sample with 750 ppm of extract, and E‐1000: sample with 1,000 ppm of extract.

According to Table 6, TVC of all samples (except E‐1000) was increased during fermentation. On day 28, the lowest TVC amount was observed in E‐1000 sample. Previous studies have also shown the effect of natural antioxidant on reducing TVC count of fermented sausages (Aksu & Kaya, 2004; Pateiro et al., 2015; Zhang et al., 2017). According to Table 6, PHE had no significant effect (*p* > .05) on TVC at the storage period.

During the fermentation period, the count of yeast and molds was increased significantly (*p* < .05) in control and E‐750 samples, but no significant change was observed in E‐500 and E‐1000 samples. On day 28, the yeast and mold count of E‐500 and E‐1000 samples was significantly (*p* < .05) lower than control sample. According to Table 6, the use of PHE had no significant effect on the reduction of the yeast and molds count during the storage period.

LAB count of all samples (except E‐1000) increased significantly (*p* < .05) during the fermentation period but had no significant change during the first month of storage (Table 6). Since LAB count of E‐1000 sample did not change significantly (*p* > .05) during the fermentation period and increased significantly during the storage period, it can be said that PHE at the concentration of 1,000 ppm can delay the growth of LAB. During the fermentation and storage period, LAB count of E‐750 sample was higher than control sample. This result was in accordance with Zhang et al. (2017) that showed the use of rose polyphenols increased LAB count of fermented sausages.

There was no significant (*p* > .05) difference in staphylococci count of all samples at the first day but PHE intensified the growth of these microorganism so that E‐750 sample had the highest staphylococci at the end of fermentation period (on day 28). After two months of storage (on day 90), lowest staphylococci were also observed in control sample and samples containing PHE had the higher counts of staphylococci (Table 6). Since staphylococci were the dominant species of starter culture used in this research, PHE addition could increase safety of product (Zhang et al., 2017).

### 3.7 Sensorial evaluation of sausage samples

According to the results of TBARS, texture, and color, E‐750 sample was better than the other two samples containing PHE. For this reason, E‐750 sample was considered as the optimum sample and its sensory characteristics were compared with the control sample. As shown in Table 7, no significant differences in taste, odor, and overall acceptability scores had among E‐750 sample and control sample. Therefore, it could be said that addition of the PHE did not change the sensory quality of the fermented sausage during storage for two months. According to this finding, Kurćubić et al. (2014) also showed that *Kitaibelia vitifolia* extract had no effect sensory scores of fermented sausage.

**Table 7:** Sensorial evaluation of sausage samples during fermentation and storage period

Treatment	Control	E‐750
Flavor	3.37 ± 0.58^a^	3.33 ± 0.31^a^
Odor	3.27 ± 0.48^a^	3.23 ± 0.25^a^
Overall acceptability	3.41 ± 0.55^a^	3.36 ± 0.56^a^

Note

Results are expressed as means ± standard deviation; different lowercase letters represent a significant difference in each row (*p* < .05).

Control: sample without any extract and E‐750: sample with 750 ppm of extract, and E‐1000: sample with 1,000 ppm of extract.

## 4 CONCLUSION

This study investigated the effect of PHE as a natural antioxidant and antimicrobial on increasing the quality of fermented sausages during the fermentation and storage period. The results showed that addition of PHE increased the L^*^ and a^*^ values of fermented sausages. Moreover, PHE affected hardness and chewiness of samples and had inhibitory effect on lipid oxidation of samples. Evaluation of microbial properties showed that PHE decreased TVC and yeast and molds of fermented sausages but had not inhibitory effect on desirable bacteria (such as LAB and staphylococci). In general, the results of this study showed that PHE as a natural antioxidant and preservative could increase the quality of fermented sausages.

## CONFLICT OF INTEREST

The authors confirm that this article content has no conflict of interest.

## ETHICAL STATEMENTS

This study does not involve any human or animal testing.
